# Expression of M30 and M65 in celiac disease. Analyticalcross-sectional study

**DOI:** 10.1590/1516-3180.2018.0241161118

**Published:** 2018-12-13

**Authors:** Evrim Kahramanoğlu Aksoy, Gülçin Güler Şimşek, Murat Torgutalp, Ferdane Pirinççi Sapmaz, Muhammet Yener Akpınar, Metin Uzman, Yaşar Nazlıgül

**Affiliations:** I MD. Gastroenterologist, Department of Gastroenterology, Keçiören Training and Research Hospital, Ankara, Turkey.; II MD. Associate Professor and Pathologist, Department of Pathology, Keçiören Training and Research Hospital, Ankara, Turkey.; III MD. Rheumatological Researcher, Department Rheumatology, Ankara University Faculty of Medicine, Ankara, Turkey.; IV MD. Associate Professor and Gastroenterologist, Department of Gastroenterology, Keçiören Training and Research Hospital, Ankara, Turkey.; V MD. Gastroenterologist, Department of Gastroenterology, Keçiören Training and Research Hospital, Ankara, Turkey.; VI MD. Associate Professor and Gastroenterologist, Department of Gastroenterology, Keçiören Training and Research Hospital, Ankara, Turkey.; VII MD. Professor and Gastroenterologist, Department of Gastroenterology, Keçiören Training and Research Hospital, Ankara, Turkey.

**Keywords:** disease. M30 cytokeratin-18 peptide, human. M65 antigen, human

## Abstract

**BACKGROUND:**

The role of villous atrophy in apoptosis, a distinctive feature of celiac disease, is a matter of controversy. The aim of this study was to determine the apoptosis rate through immunohistochemical staining for M30 and M65 in celiac disease cases.

**DESIGN AND SETTING:**

Analytical cross-sectional study in a tertiary-level center.

**METHODS:**

Duodenal biopsies from 28 treatment-naive patients with celiac disease, 16 patients with potential celiac disease, 10 patients with a gluten-free diet and 8 controls were subjected to immunohistochemical staining for the end-apoptotic marker M30 and the total cell death marker M65. *H-*scores were compared. Several laboratory parameters were recorded concomitantly, and at the one-year follow-up for celiac disease and potential celiac disease patients.

**RESULTS:**

There was a significant difference in *H*-score for M30 expression between the celiac disease, potential celiac disease and gluten-free diet groups (P = 0.009). There was no significant difference in *H*-score for M65 expression. There was a positive correlation between the *H*-score for M30 expression and the anti-tissue transglutaminase immunoglobulin A (anti-tTgIgA) and anti-tissue transglutaminase immunoglobulin G (anti-tTgIgG) levels (R = 0.285, P = 0.036; and R = 0.307, P = 0.024, respectively); and between the *H*-score for M65 expression and the anti-tTgIgA and anti-tTgIgG levels (R = 0.265, P = 0.053; and R=0.314, P = 0.021, respectively). There was no difference between celiac disease and potential celiac disease patients regarding the laboratory parameters selected.

**CONCLUSION:**

The rates of apoptosis and nutritional deficiencies in patients with potential celiac disease were similar to those in patients with celiac disease.

## INTRODUCTION

Celiac disease is an immune-mediated disease triggered by gluten exposure in genetically susceptible people.^1^ The clinical symptoms are variable, ranging from classical malabsorption symptoms to atypical presentations and asymptomatic forms that are detected incidentally during serological screening^2^^,^^3^ Serum anti-tissue transglutaminase (anti-tTg) immunoglobulin A (anti-tTgIgA), serum anti-tissue transglutaminase immunoglobulin G (anti-tTgIgG) and anti-endomysial antibodies (EmA) are widely used for serological screening.^4^ Whereas the diagnosis of celiac disease can be made among children in the presence of very high levels of anti-tTg antibodies (i.e. > 10 times above the upper normal limit), a duodenal biopsy is essential for this diagnosis among adults. Human leukocyte antigen (HLA) typing needs to be done and histological changes after a gluten-free diet need to be seen in order to confirm the diagnosis in patients who are seronegative for celiac disease.^5^^,^^6^

While mucosal villous atrophy with crypt hyperplasia (Marsh 3) is defined as celiac disease, normal mucosa (Marsh 0), intraepithelial lymphocytosis (Marsh 1) and intraepithelial lymphocytosis with crypt hyperplasia (Marsh 2) in seropositive and genetically predisposed patients are defined as cases of potential celiac disease.^7^ Although vitamin and mineral deficiencies are seen both in patients with celiac disease and in those with potential celiac disease, most clinicians only recommend a gluten-free diet for patients with celiac disease. Kurppa etal.^8^^,^^9^^,^^10^ showed the favorable effects of a gluten-free diet in terms of improvement of clinical signs, mucosal healing and antibody titer reduction.

Villous atrophy is the hallmark of celiac disease. It results from increased enterocyte destruction and deficient epithelial cell regeneration. Several studies have mentioned that apoptosis has a role in villous atrophy.^11^^,^^12^ While higher rates of apoptosis in celiac disease cases were shown in several studies, Augustin etal. were unable to demonstrate higher apoptotic activity.^11^^,^^13^^,^^14^ Das etal. indicated that the rates of apoptosis in patients with mild enteropathy and advanced enteropathy were similar.^15^

M30 is a caspase-cleaved keratin 18 (CK-18) cytoskeletal protein that is known to be a marker for the end of apoptosis. M65 comprises both cleaved and uncleaved CK-18 and is used as a marker for total cell death in situations of both necrosis and apoptosis.^16^

## OBJECTIVE

In this study, we aimed to investigate the apoptosis rate as a marker of disease severity through immunohistochemical staining for M30 and M65 in the duodenal mucosa of patients with potential celiac disease and patients with a gluten-free diet, in comparison with patients with celiac disease; and to investigate whether there might be any difference between the groups in terms of nutritional deficiency.

## METHODS

### Design and setting

This was an analytical cross-sectional study and data were collected from the laboratory and pathology archives of Keçiören Training and Research Hospital. Newly diagnosed celiac disease patients, patients with a gluten-free diet and patients with potential celiac disease who came for consultations at our gastroenterology outpatient clinic between 2010 and 2017 were included the study and compared regarding apoptosis rate in the gut. Allpatients were followed up for at least one year. Some selected laboratory parameters (hemoglobin, iron, ferritin, folate, vitamin B12, 25-hydroxy-vitamin D3, albumin, calcium, magnesium and phosphorus) were compared at diagnosis and after one year of follow-up among newly diagnosed celiac disease patients and patients with potential celiac disease.

### Ethical considerations

This study was approved by an internal review board on June 9, 2017, under the approval number 43278876-929-441-3137, and informed consent was obtained from all patients.

### Participants

The sample size was calculated using 90% power and 1% margin of error, as described in the study by Shalimar etal., which used 3 patients for villus evaluation and 28 patients for crypt evaluation.^11^ A total of 54 participants were included: 28 treatment-naive patients with celiac disease, 16 patients with potential celiac disease and 10 patients who had been using a gluten-free diet.

The diagnosis of celiac disease was made according to the presence of clinical symptoms with positive results for anti-tTgIgA or anti-tTgIgG and presence of villous atrophy (Marsh 3). All of these patients had previously been receiving a gluten-free diet since receiving their diagnoses. The diagnosis of potential celiac disease was made according to the presence of antibody seropositivity and Marsh 0, 1 or 2 criteria in pathological examinations. Thestudy also included 10 previously diagnosed celiac disease patients who had been using a gluten-free diet for 2-8 years. Sixof these patients had duodenitis and four of them had a normal appearance in endoscopic examinations. Three of these patients had borderline positive antibody serological findings and Marsh0 appearance in pathological examinations. Patients with refractory celiac disease, coexistent systemic diseases, human immunodeﬁciency virus seropositivity or positive stool tests for parasitic infections were excluded.

Eight patients with dyspepsia who underwent esophagogastroduodenoscopy were recruited as controls. The individuals included in this control group were firstly matched for age and gender. They all presented negative results from endoscopic examinations. Multiple duodenal biopsy samples were obtained from the bulbus and the second part of the duodenum. All the controls were negative for anti-tTgIgA and anti-tTgIgG. Their laboratory parameters were normal and their duodenal biopsy specimens showed normal morphology. They were diagnosed as having functional dyspepsia and it was confirmed that they were not using a gluten-free diet.

### Variables

Demographic data, serological (serum anti-tTgIgA and anti-tTgIgG levels), biochemical and hematological parameters and clinical and pathological data were obtained from the file records. Serum anti-tTgIgA and anti-tTgIgG levels were measured by means of the enzyme-linked immunosorbent assay (ELISA) (ImmuLisa, Immco, USA). Anti-tTgIgA or anti-tTgIgG antibody levels > 18 U/ml were accepted as positive and levels between 12 and 18 U/ml were accepted as borderline. Upper gastrointestinal endoscopic views of the duodenal folds were recorded (normal, attenuated and scalloped mucosal folds).

### Histological examination

Mucosal biopsies were processed in an automated tissue processor and were set into paraffinized tissue blocks. Hematoxylin and eosin (H&E)-stained slides were prepared from the biopsy samples. Biopsy specimens were considered to be sufficient if at least 3-4 crypts were seen, arranged on the muscularis mucosae. All the duodenal biopsy specimens were evaluated by the same pathologist, who was experienced in celiac disease and was unaware of the patients’ clinical data. The modified Marsh grading system described by Oberhuber was used to grade the mucosal changes for both the end of apoptosis (M30) and total cell death, i.e. apoptosis and necrosis (M65).^17^

### Immunohistochemistry

Poly-l-lysine coated slides were prepared from 4-5 µm sections from the paraffin-embedded blocks and were processed for immunohistochemical (IHC) evaluation. All of these slides were subjected to IHC staining for: 1) the end-apoptosis marker protein M30 (CytoDEATH antibody) for detection of caspase-cleaved cytokeratin 18 neo-epitope M30 (PEVIVA, Sweden; 1:100); and 2) the end-apoptosis and necrosis marker protein M65 for detection of uncleaved cytokeratin 18 (LifeSpan Biosciences, Inc., Seattle, WA, USA). A standard overnight IHC staining protocol was applied and the universal secondary antibody (REAL EnVision System, DAKO, Glostrup, Denmark) was used. 3,3-diaminobenzidine and hydrogen peroxide were used for immunostaining.

The IHC-stained sections were described in terms of the distribution and intensity of the staining of the villi in the mucosal biopsies. The intensity of marker staining was graded as follows: grade 1, pale stain expression; grade 2, moderate stain expression; or grade 3, robust stain expression. The distribution of marker staining was graded as follows: grade 0, from no staining to <10% mucosal area positivity; grade 1, 11%-25% area positivity; grade 2, 26%-60% area positivity; or grade 3, ≥ 61% area positivity. The*H*-scores for villi were calculated by multiplying together the stain distribution grade and intensity grade in a single biopsy that contained both villi and crypt epithelium. All the biopsies were examined by one experienced pathologist.

### Statistical analyses

Statistical analyses were performed using the computer software Statistical Package for the Social Sciences (SPSS), version 22.0 (IBM, Armonk, NY, USA). Normally distributed continuous variables were expressed as the mean and standard deviation, while non-normally distributed variables were expressed as the median and interquartile range (IQR). Comparisons were made using the Mann-Whitney U test or Kruskal-Wallis test if the distribution of the variables was not normal; and the t test or one-way ANOVA were used if the distribution of the variables was normal. Categorical data were presented as proportions, and the chi-square test or Fisher’s exact test was used to compare proportions in different groups. P-values < 0.05 were taken to be statistically significant.

## RESULTS

### Baseline characteristics of participants

Mucosal biopsies were obtained from 28 patients with celiac disease, 16 patients with potential celiac disease and 10 patients with a gluten-free diet; and from eight patients with dyspepsia who formed a control group for the study. The demographic data and data from some selected laboratory parameters among the patients (including those with celiac disease, potential celiac disease and gluten-free diet) are summarized in Table 1. The endoscopic findings and Marsh scores are shown in Table 2.

**Table 1.** Demographic properties and some selected laboratory parameters of the patients and control group

	Patients (n = 54)	Control (n = 8)	P
Age, years	30.4 ± 9.1	28.6 ± 5.4	0.59
Female sex, %	35 (64.8%)	5 (62.5%)	1.0
Height, cm	162.2 ± 7.5	165 ± 7.0	0.23
Weight, kg	59.4 ± 8.2	65.9 ± 10.1	0.12
BMI, kg/m^2^	22.5 ± 1.7	23.9 ± 2.1	0.09
Anti-tTgIgA (IQR) (U/ml)	147.5 (235)	2.5 (1.68)	< 0.001
Anti-tTgIgG (IQR) (U/ml)	90 (96.25)	3.34 (2.03)	< 0.001
Hemoglobin, g/dl	12.4 ± 2.1	14.4 ± 0.5	0.009
Ferritin (IQR), ng/ml	11.3 (15.6)	46.5 (14.5)	0.001
Iron, µg/dl	53.3 ± 38.9	88.6 ± 15.7	< 0.001
Vitamin B12, pg/ml	220.6 ± 85.2	270.5 ± 50.4	0.113
25OHD3, ng/ml	13.3 ± 7.2	30.7 ± 5.8	< 0.001
Folate, ng/ml	4.7 ± 2.1	7.1 ± 0.8	< 0.001
Albumin, g/dl	3.9 ± 0.3	4.2 ± 0.3	0.11
Calcium, mg/dl	8.7 ± 0.6	9.0 ± 0.3	0.037
Magnesium, mg/dl	1.9 ± 0.2	2.1 ± 0.1	0.31
Phosphorus, mg/dl	2.9 ± 0.6	2.8 ± 0.4	0.74

BMI = body mass index; Anti-tTgIgA = anti-transglutaminase immunoglobulin A antibodies; IQR = interquartile range; Anti-tTgIgG = anti-transglutaminase immunoglobulin G antibodies; 25OHD3 = 25-hydroxy-vitamin D3.

**Table 2.** Numbers of participants according to endoscopic findings and Marsh grade (P = 0.053; and R = 0.314, P = 0.021, respectively)

Numbers of participants	CD (n = 28)	PCD (n = 16)	GFD (n = 10)	Controls (n = 8)
Endoscopic findings				
Duodenitis	4	5	6	5
Attenuated duodenalfolds	11	7		
Scalloping of duodenal folds	9			
Normal duodenal folds	4	4	4	3
Marsh grade				
Marsh 0		2	10	
Marsh 1		8		
Marsh 2		6		
Marsh 3a	9			
Marsh 3b	7			
Marsh 3c	12			

CD = celiac disease; PCD = potential celiac disease; GFD = gluten-free diet.

### Expression of M30 and M65 markers

**Figure 1.**
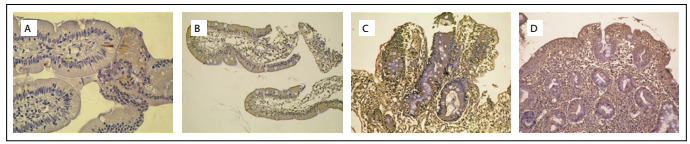
M30 staining, showing positivity of epithelial cytoplasm in the duodenal mucosa of the following patients: a) with a gluten-free diet (Marsh 0) [100 X]; b) with potential celiac disease (Marsh 1) [40 X]; c) with potential celiac disease (Marsh 2) [100 X]; and d) with celiac disease (Marsh 3c) [100 X].

**Figure 2.**
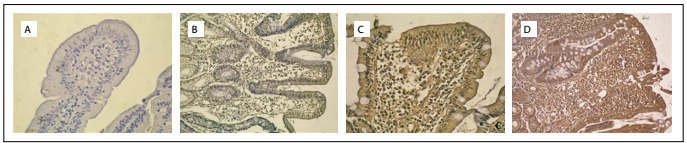
M65 staining, showing positivity of epithelial cytoplasm in the duodenal mucosa of the following patients: a) with a gluten-free diet (Marsh 0) [100 X]; b) with potential celiac disease (Marsh 1) [100 X]; c) with potential celiac disease (Marsh 2) [100 X]; and d) with celiac disease (Marsh 3c) [100 X].

There was no stain expression in the mucosal biopsies of the control group (Table 3). Altogether, there was a significant difference in *H*-score for M30 expression between the celiac disease, potential celiac disease and gluten-free diet groups (P = 0.009). However, there was no significant difference specifically between the celiac disease and potential celiac disease groups (P = 0.25). The statistical difference found in the overall group comparison was due to differences between the celiac disease and gluten-free diet groups (P = 0.001) and between the potential celiac disease and gluten-free diet groups (P < 0.001). Although there was a difference in *H*-score for M65 expression, this was not statistically significant (P = 0.053). There was only a significant difference between the potential celiac disease and gluten-free diet group (P=0.04) (Table 4; Figures 1 and 2).

**Table 3.** *H-*scores for M30 and M65 expression in participants

	Patients (n = 54)	Controls (n = 8)	P
*H-*score for M30 expression	3.8 ± 2.1	0	< 0.001
*H-*score for M65 expression	4.1 ± 2.0	0	< 0.001

**Table 4.** Comparison of *H-*scores for M30 and M65 expression between patient groups

	CD (n = 28)	PCD (n = 16)	GFD (n = 10)	P
*H*-score for M30 expression	3.9 ± 2.6	4.7 ± 1.6	2.2 ± 0.9	0.009
CD-PCD (P = 0.25) / CD-GFD (P = 0.001) / PCD-GFD (P < 0.001)				
*H-*score for M65 expression	3.9 ± 1.9	5.1 ± 2.4	3.2 ± 1.5	0.053
CD-PCD (P = 0.08) / CD-GFD (P = 0.30) / PCD-GFD (P = 0.04)				

CD = celiac disease; PCD = potential celiac disease; GFD = gluten-free diet.

### Laboratory parameters

The mean serum levels of ferritin, iron, vitamin B12, 25-hydroxy-vitamin D3, folate and calcium, and the interquartile range (IQR) levels of anti-transglutaminase antibodies for IgA and IgG, were recorded at the time when the mucosal biopsy samples were taken. There were statistically significant differences in these parameters except for the serum vitamin B12 levels between the celiac disease, potential celiac disease and gluten-free diet groups (Table5). Incomparing some selected laboratory parameters between the time when the mucosal biopsy samples were taken and one year after that time, for the celiac disease and gluten-free diet groups, there was no significant difference. There were significant differences in hemoglobin and vitamin B12 levels over the first year of follow-up (P = 0.006 and P < 0.001 respectively) (Table 6).

**Table 5.** Differences in selected laboratory parameters between patient groups

	CD (n = 28)	PCD (n = 16)	GFD (n = 10)	P
Ferritin, ng/ml	16.9 ± 17.2	18.5 ± 12.1	40.7 ± 28.2	0.003
CD-PCD (P = 0.96) / CD-GFD (P = 0.003) / PCD-GFD (P = 0.012)				
Iron, µg/dl	42.0 ± 32.1	51.4 ± 38.8	88 ± 40	0.004
CD-PCD (P = 0.68) / CD- GFD (P = 0.003) / PCD-GFD (P = 0.037)				
25OHD3, ng/ml	11.7 ± 5.1	12.3 ± 7.9	19.2 ± 8.4	0.013
CD-PCD (P = 0.68) / CD-GFD (P = 0.003) / PCD-GFD (P = 0.039)				
Folate, ng/ml	4.1 ± 1.6	4.3 ± 2.0	6.6 ± 2.3	0.003
CD-PCD (P = 0.97) / CD-GFD (P = 0.003) / PCD-GFD (P = 0.009)				
Calcium, mg/dl	8.6 ± 0.6	8.6 ± 0.6	9.39 ± 0.6	0.007
CD-PCD (P = 0.99) / CD-GFD (P = 0.017) / PCD-GFD (P = 0.008)				
Anti-tTgIgA (IQR)	300 (148)	125 (133)	45 (45)	< 0.001
CD-PCD (P = 0.007) / CD-GFD (P < 0.001) / PCD-GFD (P < 0.001)				
Anti-tTgIgG (IQR)	104 (160)	103 (78)	34 (29)	0.001
CD-PCD (P = 0.80) / CD-GFD (P < 0.001) / PCD-GFD (P < 0.001)				
Vitamin B12, pg/ml	241.2 ± 99.6	184.2 ± 57.6	184.2 ± 57.6	0.10

CD = celiac disease; PCD = potential celiac disease; GFD=gluten-free diet; 25OHD3 = 25-hydroxy-vitamin D3; anti-tTgIgA=anti-transglutaminase immunoglobulin A antibodies; IQR = interquartile range; anti-tTgIgG = anti-transglutaminase immunoglobulin G antibodies.

**Table 6.** Differences between celiac disease and potential celiac disease patients regarding some selected laboratory parameters, at the time of diagnosis and after one year of follow-up

	CD		PCD		P^*^	P^**^
	At diagnosis	After one year of follow-up	At diagnosis	After one year of follow-up		
Hgb, g/dl	12.4 ± 2.1	13.4 ± 1.5	11.4 ± 2.6	11.7 ± 1.9	0.178	0.006
Iron, µg/dl	42.0 ± 32.1	67.2 ± 35.4	51.4 ± 38.8	79.5 ± 51.3	0.418	0.402
Ferritin, ng/ml	16.9 ± 17.2	28.9 ± 23.7	18.5 ± 12.1	30.3 ± 10.1	0.746	0.799
Folate, ng/ml	4.1 ± 1.6	4.9 ± 2.1	4.3 ± 2.0	4.5 ± 1.1	0.817	0.415
Vitamin B12, pg/ml	241.2 ± 99.6	341.9 ± 69.9	184.2 ± 57.6	252.6 ± 58.3	0.043	< 0.001
25OHD3, ng/ml	11.7 ± 5.1	18.9 ± 8.9	12.3 ± 7.9	21.9 ± 7.7	0.755	0.281
^†^Albumin, g/dl	3.9 ± 0.4		3.8 ± 0.6		0.804	
^†^Calcium, mg/dl	8.6 ± 0.6		8.6 ± 0.6		0.933	
^†^Magnesium, mg/dl	1.9 ± 0.2		1.9 ± 0.2		0.845	
^†^Phosphorus, mg/dl	2.9 ± 0.6		3.0 ± 0.3		0.756	

CD = celiac disease; PCD = potential celiac disease; P^*^ = difference between CD and PCD at the time of diagnosis; P^**^ = difference between CD and PCD after one year of follow-up; ^†^differential assessment was not made because of missing data at one-year follow-up.

**Figure 3.**
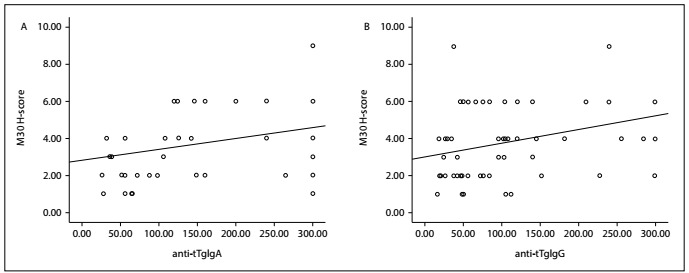
There were positive correlations between the H-score for M30 expression and: a) anti-tissue transglutaminase immunoglobulin A (anti-tTgIgA); b) anti-tissue transglutaminase immunoglobulin G (anti-tTgIgG) (R = 0.285, P = 0.036; and R = 0.307, P = 0.024, respectively).

There was a positive correlation between the *H*-score for M30 expression and the anti-tTg antibody levels for IgA and IgG (R=0.285, P = 0.036; and R = 0.307, P = 0.024, respectively). Again, there was a positive correlation between the *H*-score for M65 expression and the anti-tTg antibody levels for IgA and IgG (R=0.265, P=0.053; and R = 0.314, P = 0.021, respectively) (Figures 3 and4). There was no correlation between the *H*-scores for M30 and M65 expression and the levels of some laboratory parameters.

**Figure 4.**
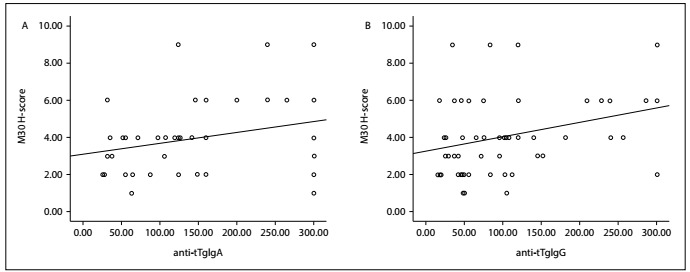
There were positive correlations between the H-score for M65 expression and: a) anti-tissue transglutaminase immunoglobulin A (anti-tTgIgA); b) anti-tissue transglutaminase immunoglobulin G (anti-tTgIgG) (R = 0.265, P = 0.053; and R = 0.314, P = 0.021, respectively).

## DISCUSSION

In the present study, we observed higher expression of the markers for both the end of apoptosis (M30) and total cell death, i.e. apoptosis and necrosis (M65), in patients with celiac disease and potential celiac disease in comparison with controls and patients with a gluten-free diet. Moreover, we did not find any significant difference in the severity of apoptosis and necrosis between patients with celiac disease and those with potential celiac disease.

There was a significant difference in nutrient deficiency levels between the patients and the control group. Comparison between the patient groups did not show any significant difference between patients with celiac disease and those with potential celiac disease regarding nutritional deficiencies. In addition, we found a positive correlation between the *H-*scores for M30 and M65 expression and the serum anti-tTg antibody levels.

Similar mucosal injuries and nutritional deficiencies were detected objectively in the patients with celiac disease and potential celiac disease in this study. The degree of micronutrient deficiencies did not correlate with the degree of apoptosis. This result was compatible with the findings of Deora etal., who showed that the degree of micronutrient deficiencies in children with celiac disease did not correlate with the degree of villous atrophy or with the serum titers of anti-tTgIgA antibodies.^17^ Zanini etal.^18^ demonstrated similar prevalences of anemia, folate deficiency, hypocholesterolemia and hypocalcemia between a group with villous atrophy and another group with mild enteropathy. Kurppa etal.^9^^,^^10^ found that symptomatic, serological and sometimes histological recovery of diseased mucosa was achieved in patients with mild enteropathy when they started using a gluten-free diet. Imperatore etal.^19^ demonstrated that asymptomatic potential celiac disease patients who continued to follow a diet containing gluten were at higher risk of developing villous atrophy and immune-mediated disorders.

It seems that potential celiac disease patients with high antibody levels (anti-tTg) present dynamic changes at the cellular level beyond what is seen through examination of the intestinal mucosa using optical microscopy. This situation may be the reason why nutritional deficiencies do not correlate with the degree of villous atrophy. Gluten-induced immune-mediated changes can be evaluated by means of apoptotic markers, even without the villous atrophy that is characteristic of celiac disease and the requirement to start using a gluten-free diet. Presence of these changes can also be used as a supportive diagnostic marker. Patients presenting such changes may need to start using a gluten-free diet.

Several previous studies have emphasized the importance of a gluten-free diet for achieving clinical and histological improvements in patients with mild enteropathy. Our findings revealed the importance of a gluten-free diet through showing that there was no difference between celiac disease and potential celiac disease patients and that there was a significant difference between patients with potential celiac disease and patients with a gluten-free diet, in terms of some selected laboratory parameters. Interestingly,the hemoglobin (13.4 ± 1.5 g/dl and 11.7 ± 1.9 ­g/­dl, respectively/ P=0.006) and vitamin B12 levels (341.9 ± 69.9 and 252.6 ± 58.3­pg/­ml, respectively/ P < 0.001) of the potential celiac disease patients were significantly lower than those of the celiac disease patients at the one-year follow-up. This may have been due to the large amount of time that was spent on challenge testing and genetic testing to ensure that an accurate diagnosis of celiac disease was made before beginning a gluten-free diet. This may also be another reason why clinicians do not emphasize the importance of a gluten-free diet to patients with potential celiac disease as much as they emphasize it to patients with celiac disease.

The most important limitation of our study was the retrospective format. Another limitation of our study was that, although there were patients with a gluten-free diet, there was no evaluation of apoptosis in mucosal biopsy specimens from the same patients after follow-up with a gluten-free diet to evaluate the changes in apoptosis and necrosis. There were no previous studies validating the use of M30 and M65 in patients with celiac disease or intestinal disease that followed a course leading to villous atrophy. Multicenter prospective studies involving large numbers of patients with celiac disease and diseases with villous atrophy other than celiac disease are needed.

## CONCLUSION

The rates of apoptosis and nutritional deficiencies in patients with potential celiac disease were similar to those in patients with celiac disease. Moreover, the apoptosis rate correlated with the anti-tTg levels. The apoptosis markers M30 and M65 may be candidate markers for celiac disease, especially for clinical follow-ups on celiac disease patients presenting histological remission.
